# Impact of Adjuvant Mannitol Administration on the Development of Delirium in Patients with Myocardial Infarction: Results of a Single-Center Pilot Study

**DOI:** 10.3390/jcm14186423

**Published:** 2025-09-11

**Authors:** Oleg Olegovich Panteleev, Maria Anatolievna Kercheva, Vyacheslav Valerievich Ryabov, Sergey Vitalievich Demianov, Evgeny Viktorovich Vyshlov

**Affiliations:** 1 Cardiology Research Institute, Tomsk National Research Medical Center, Russian Academy of Sciences, 111A Kievskaya, Tomsk 634012, Russia; kercheva@cardio-tomsk.ru (M.A.K.); rvvt@cardio-tomsk.ru (V.V.R.); svd@cardio-tomsk.ru (S.V.D.); evv@cardio-tomsk.ru (E.V.V.); 2 Cardiology Division, Siberian State Medical University, 2 Moscovsky Trakt, Tomsk 634055, Russia

**Keywords:** delirium, prevention, myocardial infarction, neuroinflammation, SIRS

## Abstract

Delirium is a severe neuropsychiatric syndrome associated with a prolonged hospital stay and adverse outcomes. **Background/Objectives:** The aim of this study was to evaluate the efficacy and safety of mannitol in patients with myocardial infarction (MI) for the prevention of delirium. **Methods:** A single-center, pilot, randomized, controlled clinical trial was conducted from 29 December 2024 to 9 May 2025. The study enrolled MI patients aged 65 years and older, with a pain-to-door time of less than 24 h, and a serum C-reactive protein (CRP) level exceeding 25 mg/L. In the mannitol group (n = 20), patients received a single 1 g/kg dose of intravenous mannitol. In the control group (n = 20), patients received standard therapy for MI. The primary outcome was the incidence of delirium. Secondary outcomes included the length of stay in the intensive care unit (ICU), the length of hospital stay, and in-hospital mortality. **Results.** The incidence of delirium in the mannitol group was 10% compared to 45% in the control group (OR = 7.3636 95% CI: 1.3372–40.5492; z = 2.294; *p* = 0.0218). The ICU LOS, in-hospital LOS, and in-hospital mortality did not differ between the two groups. **Conclusions.** Mannitol prescription for MI patients at high risk of delirium may be an effective and safe strategy for its prevention.

## 1. Introduction

Delirium is a severe neuropsychiatric syndrome, most frequently observed in elderly patients with comorbidities, associated with a prolonged hospital stay and adverse outcomes related to the underlying disease [1]. In particular, the incidence of delirium in patients with myocardial infarction (MI) leads to a prolonged length of stay (LOS) in the intensive care unit (ICU) and increased in-hospital mortality [2,3,4]. The pathogenesis of delirium has not been fully elucidated and remains controversial [5]. This fact is one of the reasons for the lack of effective methods for preventing and treating this condition [6,7,8]. Several theories address the pathophysiology of delirium: the neuroinflammatory theory, neurotransmitter deficiency theory, neuroendocrine theory, circadian dysregulation theory, and neuronal aging theory [9]. The polyetiology of delirium and the uniformity of its clinical manifestations in different nosologies suggest the existence of common processes underlying various pathogenetic pathways of delirium [10]. A previous retrospective study of delirium in patients with MI by Jäckel M et al. did not reveal any MI-specific predictors of delirium. However, a link between delirium and elevated leukocyte levels was found. Patients with delirium had higher serum CRP levels [11]. A recent meta-analysis confirmed a statistically significant link between CRP, IL 6, and TNF-α with the development of delirium [12].

The systemic inflammatory response, of which CRP is a marker, can cause endothelial dysfunction and damage to the blood–brain barrier and lead to local or generalized edema or swelling of the brain [13]. Cerebral edema is a universal non-specific response to severe injuries or infections. It is characterized by disturbances in the water–salt balance and other metabolic processes, as well as cerebral circulation disorders, which result in intra- and interstructural hyperhydration, leading to increased intracranial pressure and the exacerbation of circulatory disorders [14,15]. A retrospective study by Hamiko M. et al. [16] and a double-blind controlled study by Hemmati Maslakpak M. [17] demonstrated that the inclusion of mannitol in the composition of cardiovascular bypass prime may reduce the incidence of delirium after open-heart surgery. Similar studies have not been conducted in MI patients, which is a prerequisite for this study.

### Aim

The aim of this study is to evaluate the safety and efficacy of the administration of mannitol to patients with myocardial infarction for delirium prevention.

## 2. Materials and Methods

**Figure 1:**
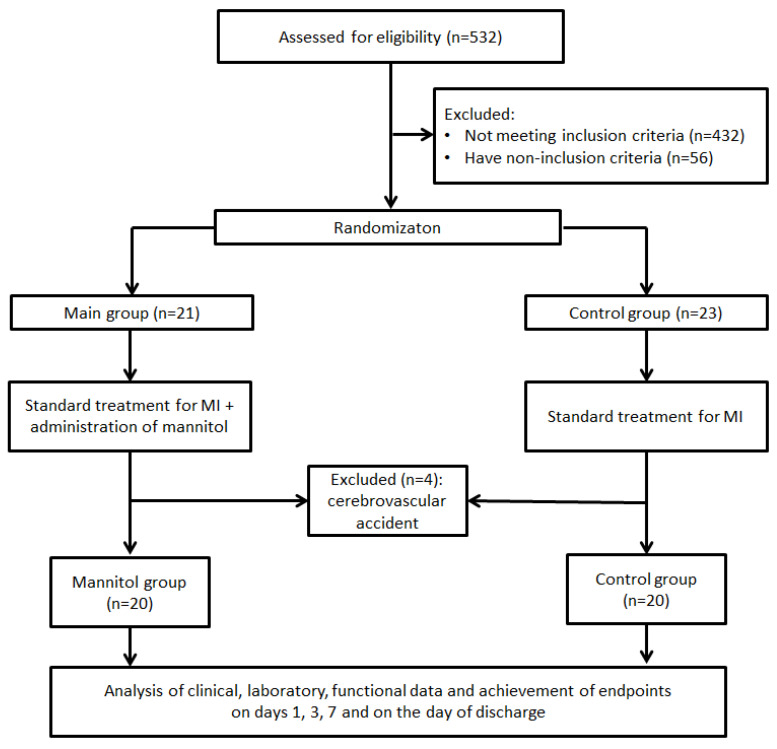
Study flow diagram.

A single-center, randomized, phase-IV pilot study was registered on Clinicaltrial.gov (NCT06759259). The study flow diagram is presented in Figure 1. The study was conducted in the Department of Emergency Cardiology of the Cardiology Research Institute, Tomsk NRMC, from 29 December 2024 to 9 May 2025.

Inclusion criteria:

1. Signing of informed consent;
2. MI within the first 24 h from onset of disease;
3. Age ≥ 65 years;
4. CRP level > 25 mg/L.

Non-inclusion criteria:

1. Documented pathological process in the brain;
2. Inability to perform the CAM-ICU test on admission;
3. Allergic reaction to mannitol;
4. Alcohol and/or drug addiction;
5. History of neuropsychiatric disorders;
6. Use of psychoactive drugs.

Exclusion criteria:

1. Acute cerebrovascular accident developed within the first 24 h of admission.

On admission, patients underwent routine anamnestic, clinical, functional, and laboratory data collection to assess eligibility for inclusion in the study. Systemic and intracardiac hemodynamic parameters were measured by echocardiography. Stroke volume (SV) was determined by Doppler imaging, taking into account the cross-sectional area of the left ventricular outlet tract (LVOT) and the integral of the linear velocity of blood flow in the LVOT. Right ventricular systolic pressure (RVSP) was determined by the integral of the linear velocity of tricuspid regurgitation during right ventricular systole, taking into account the pressure in the right atrium. The pulmonary capillary wedge pressure (PCWP) was calculated from the ratio of the mitral inflow velocity (E) to the early diastolic velocity of the mitral annulus (e’) using the Nagueh formula.

Informed consent to participate in the study was obtained from all patients.

MI was diagnosed according to the 4th universal definition [18]. Treatment for MI was performed in accordance with the 2023 ESC guidelines for the management of acute coronary syndromes [19].

Patients were randomized into two groups in a 1:1 ratio using a random number generator: the mannitol (treated with mannitol) group and the control (non-treated) group. Mannitol, due to its hyperosmolarity, is an osmotic diuretic and is an effective drug for the prevention and treatment of cerebral edema when used in a therapeutic dose (1.0–1.5 g/kg). At the same time, whether in a single dose or divided into two doses, the appointment of a therapeutic dose of mannitol can lead to hypervolemia and aggravation of the phenomena of left ventricular failure, especially in patients with myocardial infarction. Moreover, one of the complications of such a treatment regimen with mannitol can be electrolyte disorders, leading to life-threatening arrhythmias. Taking this into account, we settled on the safest method of administering mannitol as a bolus at a dose of 250 mg/kg (1/4 of the minimum therapeutic dose) followed by infusion, taking into account the clearance of mannitol at a rate of 66.6 mg/kg/h (half-life of about 90 min), until a cumulative dose of 1000 mg/kg is reached.

CAM-ICU-positive patients were defined as those who developed delirium. The test involved a two-stage assessment of the acuity and fluctuations in consciousness and attention deficit [20]. All patients underwent examination by a neurologist to exclude acute neurological pathology. When possible, CT and/or multimodal MRI + SPECT neuroimaging were performed. In cases of a positive CAM-ICU test and the absence of acute neurological pathology, patients were examined by a psychiatrist to determine the presence or absence of delirium. In patients with developed delirium, its phenotype (hyperactive, hypoactive, or mixed) was also assessed.

The primary endpoint was the incidence of delirium.

Secondary endpoints included the length of stay in the ICU, the length of hospital stay, and in-hospital mortality.

Statistical data processing was carried out using the StatTech v. 4.8.3 software package (StatTech LLC, Kazan, Russia). The conformity of quantitative data to the normal distribution was tested using the Shapiro–Wilk test. The results of the descriptive analysis are presented as arithmetic mean values (M) with standard deviation (SD). For mean values, the 95% confidence interval (95% CI) boundaries are provided. In cases of absence of normal distribution, quantitative data were presented as median (Me), and interquartile range (Q1; Q3). Categorical data are displayed as absolute values and percentages, and 95% confidence intervals for percentage values were determined using the Clopper–Pearson method. Intergroup comparisons of quantitative parameters in the presence of normal distribution were performed using Student’s t-test, provided that their variances were equal. In case of unequal variances, comparison was performed using the Welch’s T-test. The Mann–Whitney U test was used to compare groups by quantitative parameters in the absence of normal distribution. Fisher’s exact test (for expected values less than 10) was used in the analysis of contingency tables. The odds ratio with 95% confidence interval (OR; 95% CI) was calculated as the effect size when comparing relative variables. In case of zero values in the cells of the contingency table, the odds ratio was calculated using the Haldane–Anscombe correction. For the analysis of multi-way contingency tables, percentages were compared using the Pearson’s chi-square test. A predictive model for the probability of specific outcomes was developed using logistic regression. The Nagelkerke’s R^2^ coefficient served as a measure of certainty, indicating the portion of variance explained by the logistic regression model. The discriminatory ability of quantitative variables in predicting outcomes was assessed through the ROC curve analysis. The optimal cut-off value of the quantitative variable was determined by identifying the highest value of the Youden index. *p* < 0.05 was considered statistically significant.

## 3. Results

The baseline clinical and anamnestic characteristics of the patients are presented in Table 1. In the mannitol group, type 2 diabetes mellitus (DM 2) was more prevalent: 75% versus 30% (*p* = 0.01). No statistically significant differences were found for other risk factors and characteristics of MI between the groups.

No differences were observed in the main hematological, biochemical, and echocardiographic parameters between the groups (Table 2).

No significant differences in the frequency and efficacy of percutaneous coronary intervention (PCI) were found between the groups (Table 3). Conservative treatment was prescribed in three cases: two for chronic kidney disease (CKD) stages 4 and 5, and one for severe anemia. The main contraindications to the stenting of the infarction-related coronary artery (ICA) were the absence of hemodynamically significant stenoses (n = 5) and multivessel coronary disease (SYNTAX score > 32), with the inability to determine the ICA.

The study endpoints are presented in Table 4. In the main group, delirium developed less frequently: 10% versus 45% (OR = 7.3636 95% CI: 1.3372–40.5492; z = 2.294; *p* = 0.0218). In five cases (45.5%), delirium developed on the first day of hospital admission, and, in six cases (54.5%), it developed on the second day of admission. No significant difference in the frequencies of the clinical phenotypes of delirium was found. Treatment with mannitol did not reduce the ICU LOS and in-hospital LOS. In-hospital mortality did not differ between the groups. In the mannitol group, recurrent MI and cardiogenic shock were a cause of mortality in one case, and mixed genesis shock (septic and cardiogenic) led to mortality in two cases. In the control group, the fatal outcome was due to mixed genesis shock (septic and cardiogenic). No adverse events associated with mannitol administration were observed in patients from the mannitol group.

Both groups exhibited heterogeneity regarding a significant cardiovascular risk factor of type 2 diabetes; therefore, we analyzed the differences in the main clinical, laboratory, and functional parameters of patients in relation to delirium incidence. Table 5 contains the results of the analysis.

To assess the impact of clinical, laboratory, and functional parameters on delirium incidence, a number of predictive models constructed using the binary logistic regression method were analyzed. The model with the best-quality metrics determined the probability of delirium incidence based on the prophylactic administration of mannitol, the absolute neutrophil count, and the hemoglobin level. The number of observations was 40. The observed dependence is described by the following equation:

P = 1/(1 + e^−z^) × 100%

z = 3.074 − 2.301X_Mannitol_ + 0.354X_Neutrophils, n × 109/L_− 0.054X_Hemoglobin_, _g/L_

where P is the assessment of the probability of delirium incidence, z is the value of the logistic function, X_Mannitol_ is the prophylactic administration of mannitol (0—mannitol is not prescribed, and 1—mannitol is prescribed), X_Neutrophils, n × 109/L_ is neutrophils, n × 10^9^/L, and X_Hemoglobin, g/l_ is hemoglobin, g/L.

The regression model for the development of delirium is statistically significant (*p* < 0.001) in terms of the consistency between the predicted and observed values when including predictors compared to the model without predictors. The Nagelkerke’s R^2^ coefficient was 56.1%.

**Figure 2:**
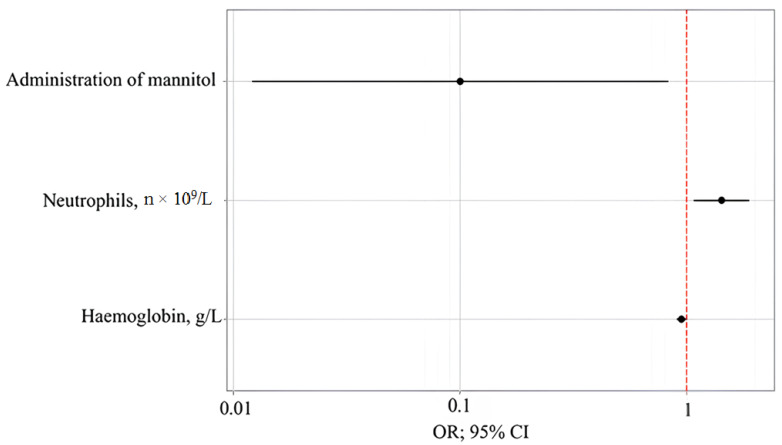
Estimates of the odds ratio with 95% CI for the predictors of delirium.

**Figure 3:**
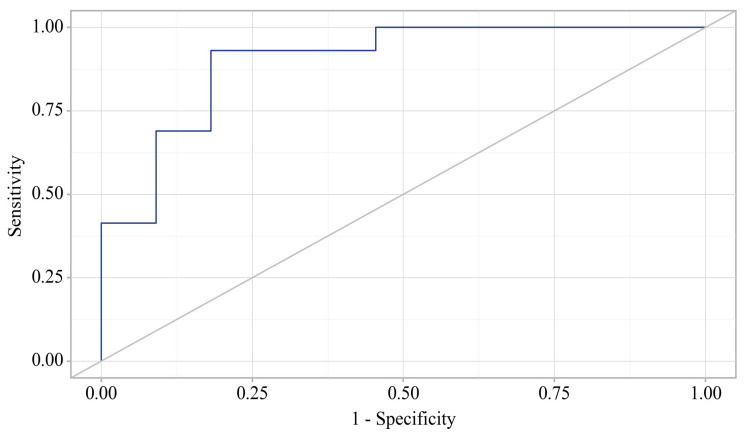
ROC curve illustrating the discriminatory ability of the regression model for the development of delirium.

**Figure 4:**
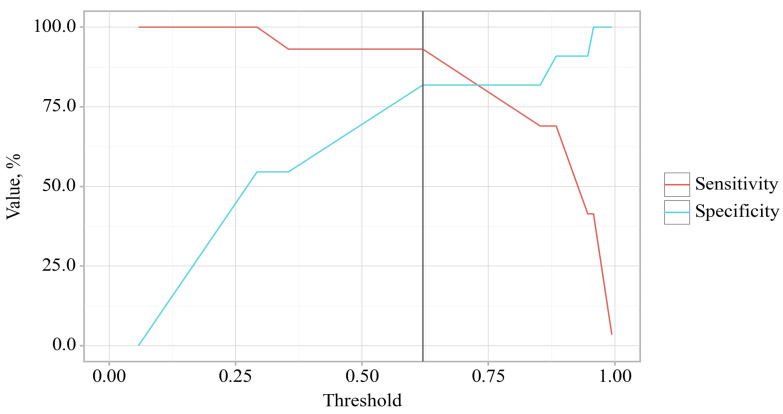
Sensitivity and specificity of the model depending on the threshold values of the expected probability of developing delirium.

With the prophylactic administration of mannitol, the incidence of delirium decreased by a factor of 9.986. With an increase in neutrophils, n × 10^9^/L by 1, the incidence of delirium increased by a factor of 1.425. With an increase in hemoglobin, g/L by 1, the incidence of delirium decreased by a factor of 1.055 (Table 6 and Table 7, Figure 2, Figure 3 and Figure 4).

The probability estimate for delirium incidence is statistically significant: AUC = 0.900; 95% CI: 0.771–1.000, *p* < 0.001.

The cut-off value of the estimated probability P, which corresponded to the highest value of the Youden index, was 0.414. The incidence of delirium was predicted at *p* values equal to or greater than this value. The sensitivity of the predictive model was 81.8%. The specificity of the predictive model was 93.1%.

## 4. Discussion

One of the main challenges in studying delirium is its polyetiology and the heterogeneity of patients enrolled in the study [21]. The inclusion criteria used allowed for a homogeneous patient population of sufficient size to address the main aim of this study. According to the literature data, the prevalence of delirium among all patients with MI is 10.9%, and, among patients with MI who are in the intensive care unit for more than 24 h, it reaches 29.7% [20]. In our study, the incidence of delirium among all patients was 27.5%, which is a comparable value given our inclusion criteria. Thus far, MI-specific risk factors for delirium have not been described. A retrospective study did not reveal associations between the phenotype of MI, its enzymatic size, and the risk for delirium [11]. Therefore, age ≥ 65 years, a known risk factor for delirium, was the only inclusion criterion in our study, along with a newly proposed criterion of CRP level > 25 mg/L. The combination of these factors has proven to be a strong predictor of delirium in MI patients: the incidence of delirium in the control group was 45%. These criteria can be used in prospective studies of MI-associated delirium to reduce the population size and optimize protocols for randomized trials.

The difference in the prevalence of type 2 diabetes in the main and control groups could hypothetically have influenced the study results. To assess the effects of DM 2 and other confounders on the study results, the collected data were further analyzed. The analysis did not support the hypothesis about the effect of DM 2 on the incidence of delirium. The inclusion of this parameter in the predictive models of delirium incidence led to a significant deterioration in their quality metrics. A previously conducted retrospective study on a larger patient population did not reveal an association between DM 2 and the incidence of delirium [11]. Differences in the prevalence of diabetes mellitus were likely stochastic in nature and, thus, did not significantly affect the study results.

One of the hypotheses regarding delirium is the neuroinflammatory theory [22]. A systemic inflammatory response accompanied by elevated levels of inflammatory mediators in the blood may contribute to the development of delirium by disrupting the integrity of the blood–brain barrier and resulting in cerebral edema [23,24,25]. In the developed predictive model, an elevated level of neutrophils increased the probability of delirium incidence by a factor of 1.425. With regard to the difference in the values of NLR, SII, SIRI, and AISI, these changes were most likely attributed to the activation of the innate immune system [26]. However, despite laboratory signs of a systemic inflammatory response, delirium did not develop in all patients enrolled in the study. This suggests that this relationship is not direct and probably depends on the characteristics of the inflammatory response, as well as genetic and epigenetic factors [27,28]. The diagnosis and treatment of the systemic inflammatory response in MI patients [29,30] are not considered in modern guidelines for the management of MI; yet, this issue requires further study.

In our study, we did not focus on studying the characteristics of the systemic inflammatory response against which delirium develops. The hematological indices of systemic inflammation differed significantly in patients with and without delirium, which may indicate the importance of the intensity of the systemic inflammatory response. However, despite this, the prophylactic administration of mannitol was effective. A similar relationship was observed in recent studies of patients undergoing cardiac surgery under artificial circulation [15,16]. Moreover, the incidence of delirium in the treatment group in these studies was significantly higher than in ours. This could be due to both the specificity of the systemic inflammatory response associated with cardiac surgery and artificial circulation, and to the significantly lower doses of mannitol in the prime. Thus, the proposed scheme for the prevention of delirium with mannitol in a therapeutic dose can probably be effectively used in patients in critical conditions accompanied by systemic inflammatory response syndrome: sepsis and septic shock, oncopathology, and other pathologies associated with systemic inflammation.

The main hypothesis of our study was that a systemic inflammatory response may lead to the increased permeability of the blood–brain barrier, local or generalized cerebral edema, and impaired neurotransmitter metabolism, which, in turn, results in the clinical manifestation of delirium. When choosing an osmotic diuretic for the prevention of cerebral edema between hypertonic crystalloid solutions and mannitol, we chose mannitol due to the possibility of its introduction into the peripheral venous bed and the absence of a risk of the demyelination of nerve fibers due to a sharp increase in plasma osmolarity. Mannitol has its drawbacks. A single administration of large doses of mannitol significantly increases the volume of circulating blood, which can lead to the appearance or worsening of the signs of left ventricular failure, which is especially significant for patients with myocardial infarction, especially with severe renal impairment. This aspect requires special monitoring, especially in patients with a compromised cardiovascular system. The movement of a large volume of fluid from the interstitial space to the intravascular space during the administration of mannitol with the subsequently increased diuresis rate can lead to electrolyte disturbances and life-threatening arrhythmias, especially against the background of an ongoing ischemia-reperfusion injury of the myocardium, as well as to hypovolemia and hemodynamic instability. The monitoring of the volume status and water–electrolyte balance should be a mandatory part of monitoring during the prophylactic administration of mannitol. However, despite these shortcomings, no cases of adverse reactions associated with mannitol were registered in patients of the main group, probably due to the dosing regimen and the administration of the minimum therapeutic dose of the drug.

In this study, delirium developed less frequently in the mannitol group compared to the control group. This effect of mannitol treatment is consistent with the results reported for cardiac surgery patients [15,16]. Mannitol is an osmotic diuretic with a dehydrating effect on organs and tissues of the body, including the brain. The antioxidant properties of mannitol have also been described [31,32]. This suggests that cerebral edema plays a pivotal role in the pathophysiology of delirium. Therefore, the diagnosis of subclinical cerebral edema is essential for differentiated mannitol treatment.

Despite the decreased incidence of delirium in the mannitol group, the ICU LOS and in-hospital LOS, as well as in-hospital mortality, did not differ between the groups. This is likely due to the small population size, which boosts the need for a large-scale study to prove a statistically significant difference. Recently, data on the clinical phenotypes of delirium and their association with the frequency of adverse disease outcomes have appeared in the literature. We were unable to evaluate the effect of mannitol on the frequency of the different phenotypes of delirium due to the small sample size and the high clinical efficacy of the method of delirium prevention with mannitol.

## 5. Conclusions

Mannitol prescription in patients with myocardial infarction and an elevated serum C-reactive protein level may be an effective and safe method to prevent delirium.

### Study Limitations and Future Directions

The main limitation of the study is its single-center, non-blinded design and small sample size due to the inclusion criteria. On the one hand, this limitation does not allow us to draw comprehensive conclusions about the unconditional effectiveness of the preventive administration of mannitol to patients with myocardial infarction for the prevention of delirium. On the other hand, adding an elevated CRP level as one of the inclusion criteria allowed us to achieve an order-of-magnitude-higher incidence of delirium than in the general population, which may be useful when planning future studies to optimize the patient inclusion protocol and reduce the study sample size.

Neuroimaging in patients with myocardial infarction is associated with a number of technical and clinical difficulties. Patients with a high FiO_2_ requirement, dependent on inotropic support, an external pacemaker, and mechanical circulatory support devices, cannot be subjected to MRI neuroimaging to exclude latent infarcts in non-eloquent zones and microinfarcts. The multimodal MRI + SPECT neuroimaging of all (if possible) patients included in the study allowed us to identify and exclude from the study two patients in the control group with MRI data on the presence of microinfarcts. However, we cannot guarantee the absence of cerebral microinfarctions in patients who could not undergo MRI for various reasons. Given that delirium, according to the current definition, is considered a neuropsychiatric disorder that develops against the background of an extracerebral pathological process or systemic intoxication or hypoxia, this limitation may have a distorting effect on the observed frequency of delirium. This limitation is difficult to eliminate.

In our study, delirium in patients with myocardial infarction is associated with a systemic inflammatory response. Despite the fact that there was no significant difference in the serum CRP level in patients of both groups, we found a significant difference in the hematological indices of systemic inflammation between patients without delirium and patients with delirium. Systemic inflammatory response syndrome in patients with delirium was not considered in our study, although it apparently plays a significant role in the pathogenesis of neuroinflammation and the development of delirium. This fact is confirmed by the results of our study: despite a significant decrease in the frequency of delirium development with the appointment of mannitol, we did not observe a significant decrease in ICU and in-hospital LOS. Further studies should be aimed at studying the causes and characteristics of the systemic inflammatory response and searching for its relationships with brain damage.

## Figures and Tables

**Table 1:** Risk factors and characteristics of MI in patients from the mannitol and control groups.

Parameter	Main Group, n = 20	Control Group, n = 20	*p*
Men, n (%)	8 (40%)	11 (55%)	0.527
Age, years	75.5 [70; 79]	71 [68.75; 85]	0.533
High, m	1.66 ± 0.08	1.66 ± 0.08	0.78
Weight, kg	80.45 ± 13.29	82.15 ± 12.86	0.683
BMI, kg/m^2^	29.88 [27.75; 31.2]	28.22 [26.59; 32.07]	0.882
Hypertension, n (%)	20 (100%)	20 (100%)	1.0
Smoking, n (%)	4 (20%)	4 (20%)	1.0
DM 2, n (%)	15 (75%)	6 (30%)	0.01
Previous infarction, n (%)	13 (65%)	11 (55%)	0.748
GFR, mL/min/1,73 m^2^	64.22 ± 19.59	63.7 ± 26.28	0.943
Pain to door time, min.	246.5 [183; 445.5]	270 [137.5; 802]	0.715
STEMI, n (%)	8 (40%)	8 (40%)	1.0
Anterior MI, n (%)	11 (55%)	11 (55%)	0.342
Inferior MI, (%)	4 (20%)	4 (20%)	
MI with unspecified localization, n (%)	1 (5%)	4 (20%)	
Killip I, n (%)	10 (50%)	14 (70%)	0.191
Killip II, n (%)	1 (5%)	3 (15%)	
Killip III, n (%)	5 (25%)	2 (10%)	
Killip IV, n (%)	4 (20%)	1 (5%)	
GRACE	167.5 [148; 196]	172.5 [138.5; 190]	0.935
CRUSADE	9.9 [7.82; 9.9]	9.9 [6.12; 11.9]	0.32

**Abbreviations:** BMI—body mass index, STEMI—ST-elevation myocardial infarction, GFR—glomerular filtration rate, and DM—diabetes mellitus.

**Table 2:** Laboratory and instrumental data at admission.

Parameter	Main Group, n = 20	Control Group, n = 20	*p*
Hematology and biochemistry			
Hematocrit, %	39.1 [33.65; 41.32]	35.3 [31.75; 40.65]	0.449
Hemoglobin, g/L	129.5 [112.5; 135]	118.5 [110.5; 136]	0.607
WBCs, n × 10^9^/L	10.39 [7.95; 13.89]	10.89 [8.64; 12.55]	0.588
Platelets, n × 10^9^/L	227.93 ± 77.32	277.65 ± 106.23	0.099
ESR, mm/h	27.5 [20.25; 39.75]	28 [17.75; 39.25]	0.776
ALT, Units/L	23.5 [18.75; 33.5]	24 [13; 41.5]	0.829
AST, Units/L	22.05 [14.5; 38.25]	34.5 [22.75; 81.25]	0.079
Total bilirubin, µmol/L	9.65 [6.62; 18.12]	11.2 [8.77; 13.35]	0.387
Direct bilirubin, µmol/L	3.95 [2.98; 8.6]	4.5 [2.95; 6.75]	0.903
Glucose, mmol/L	11.44 [7.72; 14.82]	7.5 [6; 10.35]	0.144
Creatinine, µmol/L	90.5 [81; 114.75]	97,50 [82.25; 119]	0.892
Urea, mmolL	7.6 [6.7; 10]	7.3 [6.9; 9.47]	0.903
CRP, mmol/L	59.55 [40.72; 74.33]	56.6 [40.02; 101.5]	0.715
Echocardiography			
WMSI, points	1.59 [1.19; 2]	1.41 [1; 1.56]	0.113
PCWP, mmHg	17.25 [14.82; 20.7]	16.15 [13.97; 17.7]	0.144
RVSP, mmHg	33.5 [28.75; 48.5]	30 [25.75; 36]	0.155
LV EDV, ml	103.5 [74.25; 135.5]	93.5 [78.75; 121]	0.394
LV ESV, ml	50.5 [31.75; 81]	44 [33.5; 64.75]	0.57
LV EF (B), %	51 ± 14	51 ± 12	0.666
SV, mL	53.45 ± 20.18	47.4 ± 11.09	0.249
CO, L/min	4.34 ± 1.4	4.11 ± 1.4	0.614
CI, L/min/m^2^	2.3 ± 0.68	2.15 ± 0.68	0.48

**Abbreviations:** WBCs—white blood cell, ALT—alanine aminotransferase, AST—aspartate aminotransferase, SV—stroke volume, CO—cardiac output, CI—cardiac index, CRP—C-reactive protein, ESR—erythrocyte sedimentation rate, EF LV—left ventricular ejection fraction, LV ESV—left ventricular end-systolic volume, LV EDV—left ventricular end-diastolic volume, PCWP—pulmonary capillary wedge pressure, and RVSP—right ventricular systolic pressure.

**Table 3:** Invasive treatment of MI.

Parameter	Main Group, n = 20	Control Group, n = 20	*p*
Primary PCI, n (%)	17 (85%)	19 (95%)	0.605
Pharmaco-invasive reperfusion, n (%)	1 (5%)	1 (5%)	1.0
Conservative treatment, n (%)	2 (10%)	1 (5%)	1.0
Coronary stenting, n (%)	13 (65%)	10 (50%)	0.341
Blood flow before PCI according to TIMI 0/1/2/3 (%)	23.1/0/7.7/69.2	40/20/20/20	0.108
Blood flow after PCI according to TIMI 1/2/3 (%)	7.7/7.7/84.6	10/20/70	0.462

**Abbreviations:** PCI—percutaneous intervention.

**Table 4:** Study endpoints.

Parameter	Main Group, n = 20	Control Group, n = 20	*p*
Delirium, n (%)	2 (10%)	9 (45%)	0.031
Duration of delirium, hour	65.5 [41.75; 89.25]	132 [30; 165]	0.48
Hyperactive delirium, n (%)	2 (100)	5 (55.5)	0.563
Mixed delirium, n (%)	0 (0)	4 (44.5)	0.852
Hypoactive delirium, n (%)	0 (0)	0 (0)	1.0
ICU LOS, days	4 [2; 7.25]	3 [2; 8.5]	0.701
In-hospital LOS, days	9 [8; 15]	10 [8; 15]	0.87
In-hospital mortality, n (%)	3 (15%)	1 (5%)	0.605

**Abbreviations:** ICU—intensive care unit.

**Table 5:** Differences in the main clinical, laboratory, and functional parameters of patients at admission in relation to delirium incidence.

Parameter	Delirium «−» (n = 29)	Delirium «+» (n = 11)	*p*
HR, per minute	81.83 (18.82)	94.73 (13.02)	0.044
E/A	1.24 [0.82; 1.75]	0.73 [0.67; 0.92]	0.046
Basophils, %	0.4 [0.2; 0.5]	0.2 [0.2; 0.25]	0.041
Hematocrit, %	39 [33.7; 41.4]	33.5 [28.9; 36.2]	0.019
Hemoglobin, g/L	126.1 (22.15)	109.64 (18.42)	0.035
WBCs, n × 10^9^/L	10.17 (3.1)	13.57 (4.62)	0.01
Lymphocytes, %	20.15 (9.91)	12.23 (7.16)	0.021
Neutrophils, n × 10^9^/L	6.45 [5.28; 9.05]	9.68 [9.23; 11.84]	0.005
Neutrophils, %	69.69 (12.65)	79.88 (8.28)	0.018
SII	832.93 [589.53; 1269.35]	1791.72 [1229.09; 2695.99]	0.006
NLR	3.3 [2.57; 5.23]	8.99 [4.21; 11.07]	0.014
SIRI	2.77 [1.89; 3.75]	6.03 [3.8; 8.72]	0.041
AISI	715.84 [488.45; 1278.58]	1494.22 [765.78; 2131.09]	0.022
Mannitol, n (%)	18 (62.1)	2 (18.2)	0.031
Coronary stenting, n (%)	19 (65.5)	3 (27.3)	0.04
MRA, n (%)	19 (65.5)	3 (27.3)	0.04
Oxygen therapy, n (%)	13 (44.8)	10 (90.9)	0.012

**Abbreviations:** AISI—aggregate index of systemic inflammation calculated as Neu × Mon × Plt/Lymph, E/A—the ratio of the early peak diastolic velocity (E) to the late peak velocity in the diastolic phase (A), HR—heart rate, MRA—mineralocorticoid receptor antagonists, NLR—neu/lymph ratio, SII—systemic inflammation index calculated as Neu × Plt/Lymph, SIRI—systemic inflammatory response index calculated as Neu × Mon/Lymph, and WBCs—white blood cells.

**Table 6:** Relationships between model predictors and delirium incidence.

Predictors	Unadjusted		Adjusted	
	COR; 95% CI	*p*	AOR; 95% CI	*p*
Administration of mannitol	0.136; 0.025–0.748	0.022	0.100; 0.012–0.829	0.033
Neutrophils, n × 10^9^/L	1.307; 1.049–1.627	0.017	1.425; 1.075–1.889	0.014
Hemoglobin, g/L	0.964; 0.931–1.0	0.051	0.947; 0.901–0.997	0.036

**Table 7:** Analysis of the discriminatory ability of the estimated probability of delirium incidence.

Threshold	Sensitivity (Se), %	Specificity (Sp), %	PPV	NPV
0.747	54.5	100	100	68.8
0.702	54.5	93.1	88.8	67.2
**0.414**	**81.8**	**93.1**	**92.2**	**83.7**
0.22	81.8	69	72.5	79.1
0.148	90.9	69	74.6	88.4

## Data Availability

The data presented in the study may be requested from the corresponding author upon reasonable request.
